# 

**Published:** 1884-07

**Authors:** 


### BOOKS AND PAMPHLETS.

---

BOOKS AND PAMPHLETS RECEIVED.

*Manual of Practical Hygiene;* Parkes; vol. ii. Wm. Wood & Co.

*Medical Ethics;* Hamilton, Birminghams & Co.

*Eczema and Its Management;* Bulkley. Putnam's.

*Da Costa's Medical Diagnosis;* sixth edition.

*A Yearbook of Surgery;* Charles H. Knight. Putnam's Sons.

*Medical Education.* W. T. Keener.

*Shakespeare as a Physician;* Chesney. Chambers & Co.

*Ophthalmology for the General Practitioner;* Adolf Alt. J. H. Chambers & Co.

*Sexual neurasthenia;* George M. Beard. E. B. Treat, New York.

*Die Diphtheria. Ihre eutstehung, verhutung, und Behandlung;* Dr. Med. C. G. Rothe, Leipzig. A. Abel.

*Harper's Physicians' Vade Mecum;* Wm. Wood & Co., 1884. W. T. Keener.

*A Text-Book of Pathological Anatomy and Pathogenesis;* sec. i to viii; Ernst Ziegler. McMillan & Co. W. T. Keener.

*Hand-book of Vertebrate Dissection;* part iii. McMillan & Co., 1884. W. T. Keener.

*Pathology, Diagnosis, and Treatment of Diseases of the Rectum and Anus;* Chas. B. Kelsey.

*Wood's Library of Standard Medical Authors.* 1884. W. T. Keener.

*Practical Manual of Obstetrics;* Dr. E. Verrier.

*Wood's Library of Standard Medical Authors.* 1884. W. T. Keener.

*One Hundredth Anniversary of the Foundation of the Medical School of Harvard University.*

*Congenital sipoma, Jacobi.*

*Proceedings of the Naval Medical Society.*

*Removal of the Ovaries and Fallopian Tubes;* Batty-Tait Operation. Thomas H. Hawkins. 1884.

*Transactions of the American Ophthalmological Association.* 1883.

*Some Recent Progress in Diseases of the Nervous System;* Talbot Jones, M. D.

*The Treatment of Habitual Constipation;* Henry N. Josselyn.

*Practice at the County Infirmary;* A. W. Hagenbach.

*The Symptoms and Diagnosis of Malaria in Children;* S. Emmett Holt.

*Lister's System of Anti-Septic Wound Treatment Versus Its Modifications;* B. A. Watson.

*Annual Address Before the American Academy of Medicine;* Henry O. Macey.

*Suicide from a Medico-Legal Standpoint;* J. E. McNeill.

*Uterine and Ovarian Irritation a Cause of Insanity;* E. S. Dalberg-Norred.

*Timely Cathesis;* Thomas H. Reynolds.

*The Reciprocal Attitude of the Medical Profession and the Community;* Alexander Hutchins.

*Alcohol as a Food, a Medicine, a Poison, and a Luxury;* Geo. C. Pitzer.

*The Opium Psycho-Neurosis, Chronic Meconism or Papaverism;* C. H. Hughes.

